# Novel Mathematical Model of Breast Cancer Diagnostics Using an Associative Pattern Classification

**DOI:** 10.3390/diagnostics10030136

**Published:** 2020-03-01

**Authors:** Raúl Santiago-Montero, Humberto Sossa, David A. Gutiérrez-Hernández, Víctor Zamudio, Ignacio Hernández-Bautista, Sergio Valadez-Godínez

**Affiliations:** 1 Tecnológico Nacional de México/Instituto Tecnológico de León, León 37290, Guanajuato, Mexico; rsantiago66@gmail.com (R.S.-M.); v.zamudio@itleon.edu.mx (V.Z.); 2 Instituto Politécnico Nacional (CIC), CD de México 07738, Mexico; hsossa@cic.ipn.mx; 3 Tecnológico de Monterrey, Campus Guadalajara, Zapopan 45138, Jalisco, Mexico; 4 Cátedra-CONACyT-Tecnológico Nacional de México/I.T. León, León 37290, Guanajuato, Mexico; ihernandezb@conacyt.mx; 5 Universidad Humani Mundial, Campus San Francisco del Rincón, San Francisco del Rincón 37378, Guanajuato, Mexico; svaladezg@gmail.com

**Keywords:** breast cancer detection (BCD), associative memory (AM), associative processing (AP), neural network (NN), pattern recognition (PR)

## Abstract

Breast cancer is a disease that has emerged as the second leading cause of cancer deaths in women worldwide. The annual mortality rate is estimated to continue growing. Cancer detection at an early stage could significantly reduce breast cancer death rates long-term. Many investigators have studied different breast diagnostic approaches, such as mammography, magnetic resonance imaging, ultrasound, computerized tomography, positron emission tomography and biopsy. However, these techniques have limitations, such as being expensive, time consuming and not suitable for women of all ages. Proposing techniques that support the effective medical diagnosis of this disease has undoubtedly become a priority for the government, for health institutions and for civil society in general. In this paper, an associative pattern classifier (APC) was used for the diagnosis of breast cancer. The rate of efficiency obtained on the Wisconsin breast cancer database was 97.31%. The APC’s performance was compared with the performance of a support vector machine (SVM) model, back-propagation neural networks, C4.5, naive Bayes, k-nearest neighbor (k-NN) and minimum distance classifiers. According to our results, the APC performed best. The algorithm of the APC was written and executed in a JAVA platform, as well as the experimental and comparativeness between algorithms.

## 1. Introduction

Breast cancer is a disease in which a highly malignant type of tumor originates in breast cells. A tumor is an abnormal mass of body tissue. Tumors can be cancerous (malignant) or non-cancerous (benign). In general, tumors occur when cells divide and multiply excessively in the body. Normally, the body controls the division and growth of cells. New cells are created to replace old ones or to perform new functions. Cells that are damaged or are no longer needed die to give way to healthy replacement cells. If the balance of cell division and death is disturbed, a tumor may form. Breast cancer can be of the invasive or non-invasive type, and can occur in both men and women, although in men it is a hundred times less common than in women [1]. The risk factors for developing breast cancer are many. The most important factor is related to gender, followed by age, obesity, physical activity, diet, alcohol consumption [2] and vitamin D concentration. Although vitamin D has emerged as a potentially important determinant of breast cancer, information is still scarce. Some studies show that it can be a risk factor [3,4,5,6,7,8], while others have shown that it is not [9,10,11,12]. To date, the exact reasons for breast cancer development are unknown [1].

Worldwide, every twenty seconds a new case of breast cancer is diagnosed. Only 10% of the cases are detected at initial stages [13]. Breast cancer is the second leading cause of death in women and this number is increasing [14]. For example, in terms of U.S.A. statistics, about 1 in 8 U.S.A. women (about 12%) will develop invasive breast cancer over the course of her lifetime. In 2020, an estimated 276,480 new cases of invasive breast cancer are expected to be diagnosed in women in the U.S.A., along with 48,530 new cases of non-invasive (in situ) breast cancer. About 2620 new cases of invasive breast cancer are expected to be diagnosed in men in 2020. A man’s lifetime risk of breast cancer is about 1 in 883. About 42,170 women in the U.S.A. are expected to die in 2020 from breast cancer. Death rates have been steady in women under 50 since 2007 but have continued to drop in women over 50. The overall death rate from breast cancer decreased 1.3% per year from 2013 to 2017. These decreases are thought to be the result of treatment advances and earlier detection through screening [15,16,17,18].

A successful diagnosis in the early stages of breast cancer allows for better treatment, thereby increasing the probability of the person’s survival. The cost of breast cancer treatment is high, especially at advanced stages of the disease due to the late diagnosis [19,20].

Mammography is the most commonly used method for the diagnosis and detection of breast cancer but has several disadvantages [21]. One disadvantage is that up to 20% of false negative results are obtained from the tests. Also, false positive results are directly dependent on the radiologist´s opinion. There is also a risk of over-diagnosis which results in an excess of treatment. Mammograms require a small amount of radiation exposure that, if done repeatedly, could provoke cancer [22,23,24].

Another widely used method for the diagnosis of cancer is fine needle aspiration cytology (FNAC) [25]. The procedure of this method consists of extracting, through a needle, a sample of blood from the area affected by the cancer, and then analyzing it under a microscope. Then, according to the different characteristics of the cells, the specialist must decide whether the cancer cells are malignant or benign. However, this decision is not easy to make, and you usually choose to get a second opinion. In addition, computer information processing takes time, which leads to a demanding computational expense.

The area of computer science that is used to make an automatic classification is pattern recognition. Two of the main tasks in pattern recognition are classification and prediction. Among the most current and widespread techniques are artificial neural networks. These techniques are inspired by the behavior of biological neurons, simulating the process of their learning process. This computational model requires a set of descriptions of the classes or types to classify. The set of descriptions should be labeled to generalize the classification process [26,27,28,29,30,31].

Many methods for the diagnosis of breast cancer have been described in the literature. In [32], for example, the authors introduce a method based on associative memories for medical diagnosis including the diagnosis of breast cancer. In [33], the authors present a comparative study between several training methods of neural networks with the same objective: diagnosis of breast cancer. In [34], the authors describe another algorithm with the objective of combining a set of association rules and an artificial neural network. In [35], the researchers describe two methods, analyzed in artificial neural networks, for the diagnosis and prognosis of breast cancer. In [36], the authors combine neural networks and decision trees to solve the same problem. In summary, in [37], the researchers propose an evolutionary algorithm applied to the diagnosis of breast cancer.

In this article, we describe a classification method and use a set of numerical descriptions of patients with and without cancer. This process could help a specialist make decisions about the diagnosis of breast cancer or bi-class classification tasks in general. The simplicity of APC operations allows rapid classification to be applied to massive databases or applied in real-time processes. In addition, it does not require any prior processing of the database to extract important features. The classifier does not need to be trained with an extensive or balanced database. As we will see, a few samples (less than 10%) are sufficient to obtain a well-trained classifier with good results. Noise tolerance is another notable feature of the classifier. The algorithm generates two decision regions where, up to now, the most distorted versions of a given pattern are classified without any problem, provided they do not fall into the neutral zone generated by the APC.

## 2. Theoretical Description

Classes are natural states of objects associated with concepts [29]. We will use the letter *m* to define the number of classes denoted as $\left\{c_{i}\in \Omega |i=1,2,\ldots ,m\right\}$, where Ω is the set of all classes, known as the interpretation space. Features by which objects are characterized are known as the space representation. The goal of supervised classification is to find an inductive hypothesis in the representation space that corresponds to the structure of the interpretation space [38]. In other words, the goal is to find a pattern classifier algorithm that allows the division of the interpretation space into different regions, so that the set of known patterns can be separated in the n-dimensional space and unknown patterns can be classified. It has been shown that this can be done using associative memories. Associative memories allow pattern classification by associating them with a class or a region.

### 2.1. Associative Memories

An associative memory is a single layered neural network that allows researchers to map input patterns $x^{k}$ to output patterns $y^{k}$, such that each pattern $x^{k}$ is associated with a pattern and $y^{k}$ [39]. The formulas $x^{k}\in X^{n}\;\forall k\;\in \left\{1,2,\ldots ,p\right\}$, $y^{k}\in Y^{m}\;\forall k\;\in \left\{1,2,\ldots ,p\right\}$, and *k* are an index that represents a specific pair of associated patterns: *n* and *m* are the dimensionality of $x^{k}$ and $y^{k}$, respectively; *p* is the cardinality of the set of patterns; and **X** and **Y** are any two sets. An associative memory *M* can be represented as follows:

(1) $$x^{k}\to M\to y^{k}$$

Memory *M* is a correlation matrix of the *p* associations [40], whose fundamental set of associations is represented as:

(2) $$S=\left\{\left(x^{k},y^{k}\right)|k=1,2,\ldots ,p\right\}$$

During the learning process of memory *M*, each pair $\left(x^{k},\;y^{k}\right)\in S\left(x^{k},\;y^{k}\right)\in S$ is presented with the associative memory. During the recovery process, an input pattern $x^{\omega }$ is presented with the input of the already trained memory *M*. If $x^{k}=y^{k}$ for all k∈{1,2,…,p}, then the associative memory operates in an auto-associative way; otherwise, if, for at least one k, $x^{k}\neq y^{k}$, then the memory operates in hetero-associative way [41].

### 2.2. Associative Classification of Patterns

In [42,43,44], the authors propose an APC that combines the learning association rule of Anderson–Kohonen–Nakano´s linear associator (LA) [41,45,46] and the recovery rule of the Lernmatrix (LM) [47,48]. An APC has two advantages over an LA and an LM: (1) An APC classifier allows operation with real-valued vectors, eliminating the disadvantage of the Lernmatrix classifier that operate only with binary-valued vectors; and (2) APCs remove the orthogonality restriction on the fundamental set *S* of the linear associator [49], as well as the restriction that the number *p* of patterns of the fundamental set is small with respect to the dimension *n* of the input patterns $x^{k}$ [50,51]. It is worth mentioning that the minimum size for the training set at which an APC’s performance is stable is about 10% the size of the class with the smallest number of instances [52]. The following are given:

1. A fundamental set of associations:
  (3) $$S=\left\{\left(x^{k},y^{k}\right)|k=1,2,\ldots ,p\right\}$$
  where $x^{k}\in {\mathbb{R}}^{n}$ is the set of input patterns, $y^{k}\in {\left\{0,\;1\right\}}^{m}$ is the set of output patterns, *n* is the dimension of $x^{k}$, *m* is the dimension of $y^{k}$, and *p* is the cardinality of *S*.
2. The class c∈{1,2,…,m} to which each input pattern $x^{k}$ belongs is defined as:
  (4) $$y_{j}^{k}=\{\begin{array}{ccc} 1\; & for\;j=c & \\ 0\; & for\;j=1,2,\ldots ,c-1,c+1,\ldots ,m & \end{array}\forall k\in \left\{1,2,\ldots ,p\right\}$$

The steps for learning the APC are as follows:

1. Compute the average vector as
  (5) $$\bar{x}=\frac{1}{p}\overset{p}{\underset{k=1}{\sum }}x^{k}$$
2. Translate all the patterns of the fundamental set with respect to the mean vector as
  (6) $$x_{t}^{k}=x^{k}-\bar{x}$$
3. Build matrix M as
  (7) $$M=\overset{p}{\underset{k=1}{\sum }}y^{k}{\left[x_{t}^{k}\right]}^{t}$$

For recovery by means of the APC, the below steps should be followed (given the key pattern $x^{\omega }\in {\mathbb{R}}^{n}$).

1. Translate $x^{\omega }$ as
  (8) $$x_{t}^{\omega }=x^{\omega }-\bar{x}$$
  Perform the following product
  (9) $$z^{\omega }=Mx_{t}^{\omega }$$
2. Compute the components of class vector $y^{\omega }\in {\left\{0,1\right\}}^{m}$ as
  (10) $$y_{j}^{\omega }=\{\begin{array}{cc} 1 & \;if\;z_{j}^{\omega }=V_{h=1}^{p}z_{h}^{\omega }\\ 0 & otherwise \end{array}$$

Finally, find the index class to which $x^{\omega }\in {\mathbb{R}}^{n}$ belongs as the position *j* in vector $y_{j}^{\omega }$, where $y_{j}^{\omega }=1$.

### 2.3. Numerical Example

To understand the operation of the APC a numerical example is given next. Suppose we are given the following set of associations:

$$x^{1}=\left(\begin{array}{c} 6\\ 5\\ 2 \end{array}\right),y^{1}=\left(\begin{array}{c} 1\\ 0 \end{array}\right);\;\;\;\;\;\;\;\;\;x^{2}=\left(\begin{array}{c} -4\\ 11\\ -8 \end{array}\right),y^{1}=\left(\begin{array}{c} 0\\ 1 \end{array}\right).$$

In this case *p* = 2, *n* = 3, and *m* = 2.

Construction of the association matrix is according to the discussed material.

1. Computation of the average vector is
  $$\bar{x}=\frac{1}{p}\overset{p}{\underset{k=1}{\sum }}x^{k}=\frac{1}{2}\left[\left(\begin{array}{c} 6\\ \begin{array}{c} 5\\ 2 \end{array} \end{array}\right)+\left(\begin{array}{c} -4\\ \begin{array}{c} 11\\ -8 \end{array} \end{array}\right)\right]=\left(\begin{array}{c} 1\\ \begin{array}{c} 8\\ -3 \end{array} \end{array}\right)$$
2. Translation of the input patterns is
  $$x_{t}^{1}=x^{1}-\bar{x}=\left(\begin{array}{c} 6\\ \begin{array}{c} 5\\ 2 \end{array} \end{array}\right)-\left(\begin{array}{c} 1\\ \begin{array}{c} 8\\ -3 \end{array} \end{array}\right)=\left(\begin{array}{c} 5\\ \begin{array}{c} -3\\ 5 \end{array} \end{array}\right),\;x_{t}^{2}=x^{2}-\bar{x}=\left(\begin{array}{c} -4\\ \begin{array}{c} 11\\ -8 \end{array} \end{array}\right)-\left(\begin{array}{c} 1\\ \begin{array}{c} 8\\ -3 \end{array} \end{array}\right)=\left(\begin{array}{c} 5\\ \begin{array}{c} -3\\ 5 \end{array} \end{array}\right).$$
3. Construction of matrix *M* is
  $$M=\overset{p}{\underset{k=1}{\sum }}y^{k}{\left[x_{t}^{k}\right]}^{t}=\left(\begin{array}{c} 1\\ 0 \end{array}\right)\left(5\;-3\;\;5\right)+\left(\begin{array}{c} 0\\ 1 \end{array}\right)\left(-5\;\;3\;-5\right)=\;\left(\begin{array}{ccc} 5 & -3 & 5\\ -5 & 3 & -5 \end{array}\right)$$

Classification of the input pattern is $x=\left(\begin{array}{c} 6\\ 5\\ 2 \end{array}\right)$. We can see that this vector is a non-distorted version of vector $x^{1}$.

1. Translation of the vector is
  $$x_{t}^{1}=x^{1}-\bar{x}=\left(\begin{array}{c} 6\\ \begin{array}{c} 5\\ 2 \end{array} \end{array}\right)-\left(\begin{array}{c} 1\\ \begin{array}{c} 8\\ -3 \end{array} \end{array}\right)=\left(\begin{array}{c} 5\\ \begin{array}{c} -3\\ 5 \end{array} \end{array}\right)$$
2. The product is gotten by
  $$z^{\omega }=Mx_{t}^{\omega }=\left(\begin{array}{ccc} 5 & -3 & 5\\ -5 & 3 & -5 \end{array}\right)\left(\begin{array}{c} 5\\ \begin{array}{c} -3\\ 5 \end{array} \end{array}\right)=\left(\begin{array}{c} 59\\ -59 \end{array}\right)$$
3. Computation of the class vector $y^{\omega }$ is
  $$y^{\omega }=\left(\begin{array}{c} 1\\ 0 \end{array}\right)$$
4. The index class of vector x is found according to the above discussion. Vector $x=\left(\begin{array}{c} 6\\ 5\\ 2 \end{array}\right)$ should be classified into class number one.

Suppose we are now given a distorted version of the first vector as follows: $x=\left(\begin{array}{c} 4\\ 7\\ -1 \end{array}\right)$. Let us find again the class in which this vector should be put.

1. Translation of the vector is
  $$x_{t}^{2}=x^{2}-\bar{x}=\left(\begin{array}{c} 4\\ \begin{array}{c} 7\\ -1 \end{array} \end{array}\right)-\left(\begin{array}{c} 1\\ \begin{array}{c} 8\\ -3 \end{array} \end{array}\right)=\left(\begin{array}{c} 3\\ \begin{array}{c} -1\\ 2 \end{array} \end{array}\right)$$
2. The product is gotten by
  $$z^{\omega }=Mx_{t}^{\omega }=\left(\begin{array}{ccc} 5 & -3 & 5\\ -5 & 3 & -5 \end{array}\right)\left(\begin{array}{c} 3\\ \begin{array}{c} -1\\ 2 \end{array} \end{array}\right)=\left(\begin{array}{c} 28\\ -28 \end{array}\right)$$
3. Computation of the class vector $y^{\omega }$ is
  $$y^{\omega }=\left(\begin{array}{c} 0\\ 1 \end{array}\right)$$
4. The index of the class of vector **x** is found according to the above discussion. Vector $x=\left(\begin{array}{c} 4\\ 7\\ -1 \end{array}\right)$ should be classified into class number one.

From this very simple example, note the case of two class problems.

1. Learning and translation of the two input vectors provokes that they become the negative of each other. This is $x^{2}=-x^{1}$. Due to the fact that the output vectors for the two classes are orthogonal, matrix *M* will be composed of $x^{1}$ and its negative. This is $M=\left(\begin{array}{c} x^{1}\\ -x^{1} \end{array}\right)$. Note that between the two vectors there is a *neutral position*; this corresponds to vector $x={\left(0\;0\ldots 0\right)}^{t}$.
2. Classification of a non-distorted version of any of the input vectors’ translation provokes that it is first transformed to its translated original version. Multiplication of the association matrix *M* will always give a maximum value at the index class of the input vector.
3. Classification of a distorted version of any of the input vectors’ translation provokes that it be first moved to one of the translated original versions. The moved vector could appear on one side or the other side of its corresponding translated original version. While the noise added to the input vector does not cause that its translated version does not surpasses the neutral position, the input vector will always be correctly classified. Of course, if translation of the input vector produces $x_{t}^{\omega }={\left(0\;0\ldots 0\right)}^{t}$, then the class of the vector cannot be found because $z^{\omega }=Mx_{t}^{\omega }={\left(0\;0\ldots 0\right)}^{t}$.

Next, we discuss the details of the database used to test the performance of the APC. We also give a few words about the set of classifiers with which the APC is compared.

### 2.4. Wisconsin Breast Cancer Database

This database was compiled by Dr. William H. Wolberg at the hospitals of the University of Wisconsin, Madison [53]. We obtained the database from the pattern recognition database repository of the University of California, Irvine (UCI) [54]. It is a compilation of breast tumor cases compiled from 1989 to 1990 by FNAC. It contains 699 instances of which 458 (65.5%) belong to the class “benign” and 241 (34.5%) to the class “malignant”. Each event consists of 9 cytological features: (1) clump thickness, indicating grouping of cancer cells in multilayer; (2) uniformity of cell size, indicating metastasis to lymph nodes; (3) uniformity of cell shapes, identifying cancerous cells of varying size; (4) marginal adhesion, suggesting loss of adhesion, i.e., a sign of malignancy but the cancerous cells lose this property so this retention of adhesion is an indication of malignancy; (5) single epithelial cell size (SECS), if the SECS become larger, it may be a malignant cell; (6) bare nuclei, without cytoplasm coating, found in benign tumors; (7) bland chromatin, usually found in benign cells; (8) normal nucleoli, generally very small in benign cells; (9) mitoses, the process in cell division by which the nucleus divides.

Table 1 shows the range of values for each feature, valued on a scale of 1 to 10, with 1 being the closest to “benign” and 10 being the most anaplastic [53,55]. Moreover, the mean and standard deviation of each cytological characteristic is included. The classes that form the database, “benign” and “malignant” are not linearly separable. 

Before becoming publicly available, the dataset had 701 points. In January of 1989, after being revised, 2 instances from Group 1 were considered inconsistent and were removed from the dataset. Two more revisions occurred before the actual state of the dataset, both aimed to substitute values from zero to one, so the value range of the features is 1–10.

The data can be considered ‘noise-free’ [28] and has 16 missing values, which are the bare nuclei for 16 different instances, from Group 1 to 6. Table 1 is a summary of the state of the dataset used in this paper.

### 2.5. Minimum Distance Classifier

The minimum distance classifier (MDC) determines a given pattern belongs to a class by finding the nearest class in which the pattern can be put. This is normally done by computing a distance to each class representative [27,28,29].

### 2.6. Naïve Bayes

The naive Bayes (NB) is a classification algorithm that makes use of Bayes’ theories. The NB classifier assumes that the presence (or absence) of a particular class feature is not related to the presence (or absence) of any other feature, given the class variable [28,56,57].

### 2.7. K-Nearest Neighbor Classifier

The k-NN (k-nearest neighbors) [58] is a kind of minimum distance classifier, where for each class a sample is taken to establish to which class the pattern should be assigned. It is called nearest neighbor because the feature vector whose distance is less than the distance of the remaining vectors in the space of samples will determine the class in which the input vector should be put.

### 2.8. Back-Propagation

The back-propagation algorithm (BP) allows adjusting the weights of a neural network (NN) with the aim of finding a hyperplane or a set of hyperplanes that divide the interpretation space into different regions. This algorithm uses the gradient descent method to minimize the square error between the network´s output and the desired output [38]. Depending on the problem to be solved, the NN is configured with a number of connections, layers, input neurons and output neurons.

### 2.9. Support Vector Machine (SVM)

An SVM calculates a set of hyperplanes of separation in a high dimensional space. Hyperplanes have a maximum separation distance to the points (support vector) closest to them [59].

### 2.10. C4.5

This algorithm generates a decision tree for classification. It is based on its predecessor: the ID3 algorithm [28].

### 2.11. Comparison

A comparison among the six classifiers described in the previous section (MDC, NB, k-NN (k = 1, 2 and 3), BP, SVM and C4.5), and the ACP classifier was performed. We used the Wisconsin breast cancer database without removing the main attributes. Six experiments were conducted based on holdout validation as well as a validation experiment with 10 folds, both in a stratified manner. Experiments for holdout were 1%–99%, 10%–90%, 30%–70%, 50%–50%, and 70%–30% of the training-test, respectively. Each test for holdout was repeated one hundred times; an average classification performance was obtained. For 10-fold cross validation, ten repetitions were done, and an average performance was obtained. 

## 3. Experimental Results

Table 2 presents a summary of the holdout validation tests. One can see that in all cases the APC obtained the best classification performance. Its performance increases as the number of training patterns is incremented.

Table 3 shows a summary of the 10-fold test where APC obtained 97.13% correct classification. 

**Figure 1:**
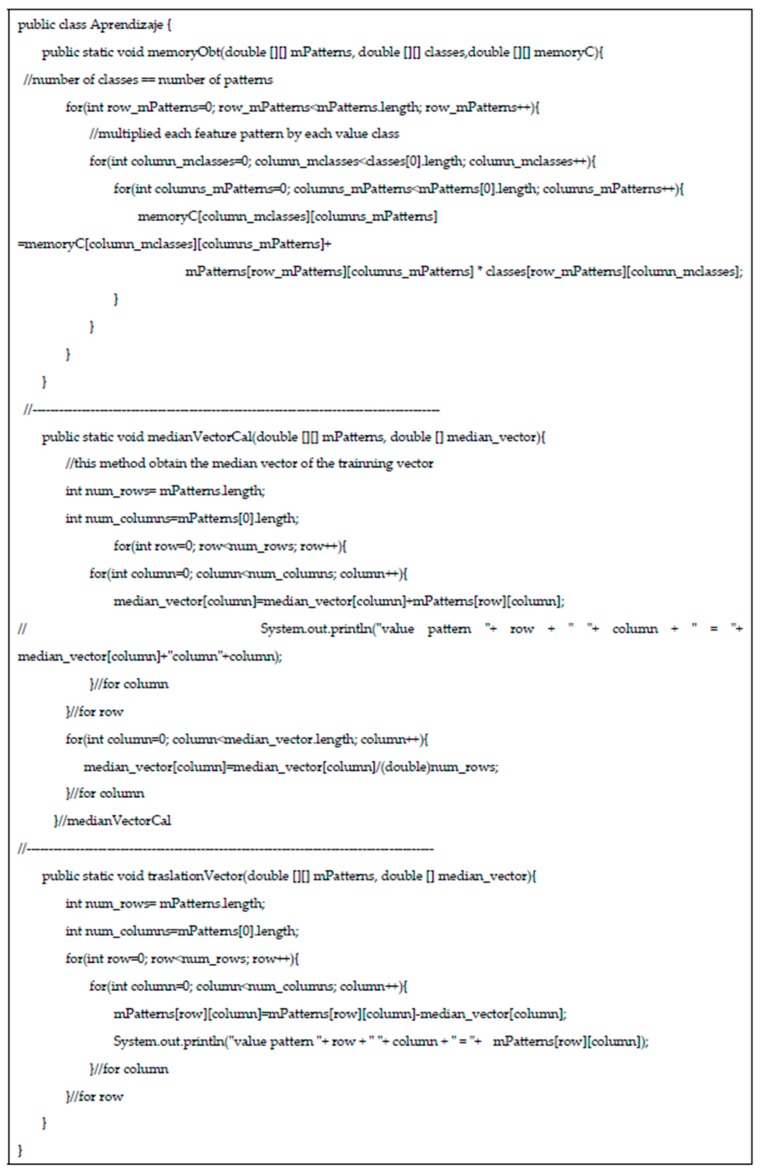
Algorithm employed for the APC classification.

Figure 1 shows the algorithm that has been used in this paper.

## 4. Conclusions

The APC classifier is a one-shot machine learning technique with low computational cost and high efficiency, with bi-class classification problems. Our technique learns with a low number of instances in each class and it is not necessary for the database to be balanced.

The APC classifier is a simple and easy to implement method that makes use of associative memories for training and testing. The diagnosis of breast cancer by associative pattern classification results in a simple and effective tool that can assist a user to make decisions concerning the prediction of breast cancer. Some methodologies that have been proposed in the literature need to extract the important features prior to training. The technique proposed in this paper does not require such a procedure. Our technique performed better than that of several well-known classifiers: support vector machines, the C4.5 algorithm based on decision trees, the naive Bayes, k-NN, and minimum distance.

Since the APC is an efficient classification algorithm in bi-class databases, it is suitable to be implemented in mobile applications can be used in short-term online diagnosis and support the process of mass population analysis. In turn, this research offers the possibility that, in the near future, software that accompanies the doctor can be developed so that, once the sample is obtained, it is characterized and included in the database being used. This would allow the specialist to obtain classification parameters on the sample and diagnose, depending on its classification, a particular breast cancer situation. This would be done quickly, backed by computer science and previously verified algorithms that offer certainty and support for decision making.

## Figures and Tables

**Table 1:** Details of the attributes of the Wisconsin database.

#	Description	Type	Range of Values	Mean	Standard Deviation
1	Clump thickness	Numeric	1–10	4.418	2.816
2	Uniformity of cell size	Numeric	1–10	3.134	3.051
3	Uniformity of cell shapes	Numeric	1–10	3.207	2.972
4	Marginal adhesion	Numeric	1–10	2.807	2.855
5	Single epithelial cell size	Numeric	1–10	3.216	2.214
6	Bare nuclei	Numeric	1–10	3.545	3.644
7	Bland chromatin	Numeric	1–10	3.438	2.438
8	Normal nucleoli	Numeric	1–10	2.867	3.054
9	Mitoses	Numeric	1–10	1.589	1.715
10	Class	Nominal	Benign, Malignant		
	Class Distribution	Benign: 458 (65.5%) Malignant: 241 (34.5%)			
	Number of missing values	16			
	Number of instances	699			

**Table 2:** Summary of holdout classification.

Training-Test			Classifier						
	ACP	MDC	NB	1-NN	2-NN	3-NN	BP	SVM	C4.5
1%–99%	96.39	94.33	92.23	95.44	88.78	91.25	66.44	94.00	93.87
10%–90%	97.16	96.06	95.60	94.96	93.52	95.69	95.52	96.20	91.93
30%–70%	97.14	95.98	96.06	95.44	94.25	96.27	96.08	96.56	93.87
50%–50%	97.31	96.05	96.15	95.62	94.84	96.55	96.37	96.75	94.32
70%–30%	97.31	96.31	96.36	95.65	95.12	96.84	96.59	96.93	94.68

**Table 3:** Summary of classification results for 10-fold cross validation.

Validation			Classifier						
	ACP	MDC	NB	1-NN	2-NN	3-NN	BP	SVM	C4.5
10-Folds	97.13	95.91	96.07	95.28	94.81	96.60	96.40	96.62	95.01
